# Skin burn related to pulse oximetry during photodynamic therapy using talaporfin sodium

**DOI:** 10.1186/s40981-018-0203-0

**Published:** 2018-09-11

**Authors:** Yuka Ino, Midori Nakashima, Tomonori Morita, Yoko Hori, Hiroaki Kishikawa, Nobutoshi Hagiwara, Takeshi Matsutani, Tsutomu Nomura, Atsuhiro Sakamoto

**Affiliations:** 1 Department of Anesthesiology, Nippon Medical School, 1-1-5 Sendagi, Bunkyo-ku, Tokyo, 113-8603 Japan; 2 Department of Anesthesiology, Sakakibara Heart Institute, 3-16-1 Asahi-cho, Fuchu-shi, Tokyo, 183-0003 Japan; 3 Department of Gastrointestinal and Hepato-Biliary-Pancreatic Surgery, Nippon Medical School, 1-1-5 Sendagi, Bunkyo-ku, Tokyo, 113-8603 Japan

**Keywords:** Photodynamic therapy, Talaporfin sodium, Skin phototoxicity, Skin burn, Pulse oximetry

## To the editor

Photodynamic therapy (PDT) is a less invasive cancer treatment [1]. One of the complications of PDT is phototoxicity because of the photosensitizer [2]. Recent clinical studies showed that no skin phototoxicity was observed during or after PDT using talaporfin sodium for esophageal cancer [3, 4].

**Fig. 1:**
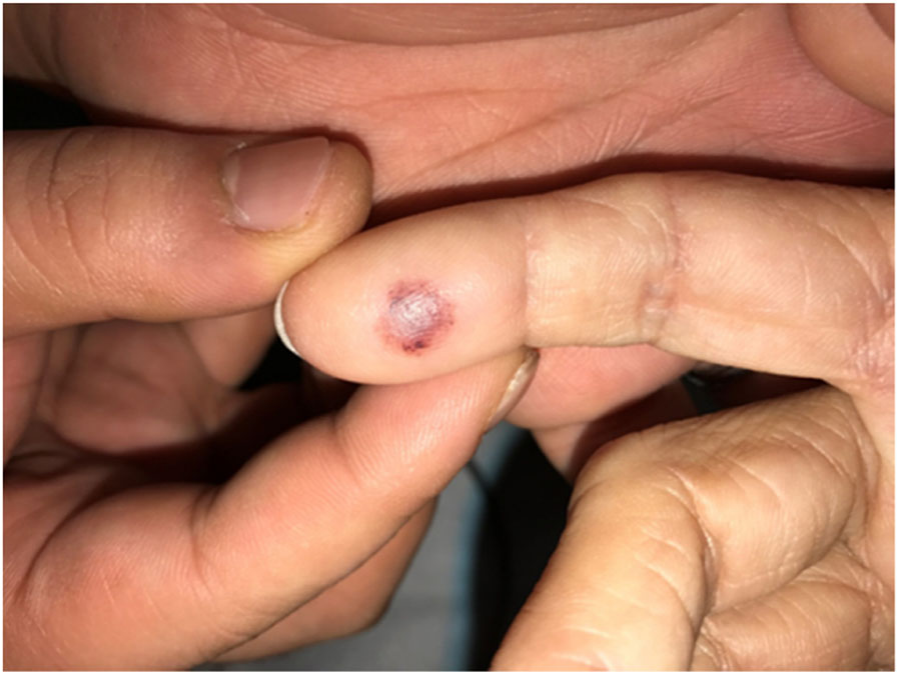
Blister on the left ring finger where the pulse oximeter had been placed during general anesthesia

A 58-year-old woman with esophageal cancer underwent esophagostomy and chemoradiation. However, the cancer grew extensively. PDT was scheduled. Talaporfin sodium was intravenously administered before PDT. We planned general anesthesia. Standard monitoring included pulse oximetry (Radical-7, Masimo Corp., Irvine, CA). We attached the pulse oximeter probes (M-LNCS Neo, Masimo Corp., Irvine, CA) to specific digits. We measured peripheral oxygen saturation for 10 min each, alternating between left and right hand. We decided to change the fingers in 10 min just from experience. The patient underwent laser beam irradiation. Semiconductor laser light of 664 nm was used. The next day, the patient showed blistering on both digits, on which the pulse oximeter probes had been applied (Fig. 1). The skin burns were treated conservatively and healed spontaneously for 2 weeks without pigmentation.

Whether PDT is performed under general anesthesia or sedation depends on each surgeon’s decision. In our institution, PDT under general anesthesia was selected for the stable operation field. The technique of pulse oximetry is based on the principle that oxygenated hemoglobin and deoxygenated hemoglobin absorb red and near-infrared light differently [5]. The MASIMO pulse oximeter probe emits red light at 660 nm and infrared light at 940 nm. Moreover, talaporfin sodium has a longer absorption at 664 nm, and the laser light of 664 nm activates talaporfin sodium as an anticancer drug [3, 4]. Both the red light emitted by pulse oximetry and the laser light are characterized by the same wavelength zone, leading us to conclude that the blister was photosensitivity dermatitis caused by the red light from the pulse oximeter. All pulse oximeter probes use similar wavelengths. Pulse oximeter sensory associated blisters must occur when using other probes. There were two previous reports of skin burns related to pulse oximetry using photofrin and mTHPC during PDT. Probes were continuously applied to patients’ fingers for 3 and 48 h [6, 7]. We predicted that shorter exposure time to red light from pulse oximetry would prevent skin phototoxicity. Nevertheless, this case showed that pulse oximetry can cause skin burns regardless of the length of pulse oximetry attachment time and the kinds of photosensitizers. Pulse oximetry is a standard monitor in anesthesia cases to detect hypoxia [5]. The World Health Organization recommends pulse oximetry for all anesthetic patients for perioperative safety [8]. There is no superior monitoring to pulse oximetry for measuring oxygenation, even during PDT. Therefore, we should monitor peripheral oxygen saturation by pulse oximetry as little as possible or intermittently. In future cases, we might need arterial cannulation to assess blood oxygenation, if we will not be able to apply pulse oximetry.

Before PDT, the anesthesiologist should inform the patient regarding the possibility of skin burns related to pulse oximetry.

## Abbreviations

mTHPC: Meta-tetrahydroxyphenylchlorin
PDT: Photodynamic therapy

## Authors’ information

YI M.D. is a Staff Anesthesiologist in the Department of Anesthesiology, Nippon Medical School Hospital; MN M.D. is a Staff Anesthesiologist in the Department of Anesthesiology, Nippon Medical School Hospital; TM M.D., PhD., is a Staff Anesthesiologist in the Department of Anesthesiology, Sakakibara Heart Institute; YH M.D., PhD., is a Staff Anesthesiologist in the Department of Anesthesiology, Nippon Medical School Hospital; HK M.D., PhD. is a Director of Anesthesiologists in the Department of Anesthesiology, Nippon Medical School Hospital; NH M.D., PhD., is a staff member in the Department of Gastrointestinal and Hepato-Biliary-Pancreatic Surgery, Nippon Medical School Hospital; TM M.D., PhD. is Associate professor of Gastrointestinal and Hepato-Biliary-Pancreatic Surgery, and Nippon Medical School Hospital; TN M.D., PhD. is Associate professor of Gastrointestinal and Hepato-Biliary-Pancreatic Surgery, and Nippon Medical School Hospital; AS M.D., PhD. is Professor and Chair of Anesthesiology, Nippon Medical School.
